# SARS-CoV-2 Delta variant induces enhanced pathology and inflammatory responses in K18-hACE2 mice

**DOI:** 10.1371/journal.pone.0273430

**Published:** 2022-08-29

**Authors:** Katherine S. Lee, Ting Y. Wong, Brynnan P. Russ, Alexander M. Horspool, Olivia A. Miller, Nathaniel A. Rader, Jerome P. Givi, Michael T. Winters, Zeriel Y. A. Wong, Holly A. Cyphert, James Denvir, Peter Stoilov, Mariette Barbier, Nadia R. Roan, Md. Shahrier Amin, Ivan Martinez, Justin R. Bevere, F. Heath Damron

**Affiliations:** 1 Department of Microbiology, Immunology, and Cell Biology, West Virginia University, Morgantown, WV, United States of America; 2 Vaccine Development Center at West Virginia University Health Sciences Center, Morgantown, WV, United States of America; 3 Department of Pathology, Anatomy, and Laboratory Medicine, West Virginia University, Morgantown, WV, United States of America; 4 Department of Biological Sciences, Marshall University, Huntington, WV, United States of America; 5 Department of Biomedical Sciences, Marshall University, Huntington, WV, United States of America; 6 Department of Biochemistry, School of Medicine, West Virginia University Morgantown, Morgantown, WV, United States of America; 7 Department or Urology, University of California, San Francisco, San Francisco, CA, United States of America; 8 Gladstone Institute of Virology, San Francisco, CA, United States of America; 9 West Virginia University Cancer Institute, School of Medicine, Morgantown, WV, United States of America; University of Minnesota College of Veterinary Medicine, UNITED STATES

## Abstract

The COVID-19 pandemic has been fueled by SARS-CoV-2 novel variants of concern (VOC) that have increased transmissibility, receptor binding affinity, and other properties that enhance disease. The goal of this study is to characterize unique pathogenesis of the Delta VOC strain in the K18-hACE2-mouse challenge model. Challenge studies suggested that the lethal dose of Delta was higher than Alpha or Beta strains. To characterize the differences in the Delta strain’s pathogenesis, a time-course experiment was performed to evaluate the overall host response to Alpha or Delta variant challenge. qRT-PCR analysis of Alpha- or Delta-challenged mice revealed no significant difference between viral RNA burden in the lung, nasal wash or brain. However, histopathological analysis revealed high lung tissue inflammation and cell infiltration following Delta- but not Alpha-challenge at day 6. Additionally, pro-inflammatory cytokines were highest at day 6 in Delta-challenged mice suggesting enhanced pneumonia. Total RNA-sequencing analysis of lungs comparing challenged to no challenge mice revealed that Alpha-challenged mice have more total genes differentially activated. Conversely, Delta-challenged mice have a higher magnitude of differential gene expression. Delta-challenged mice have increased interferon-dependent gene expression and IFN-γ production compared to Alpha. Analysis of TCR clonotypes suggested that Delta challenged mice have increased T-cell infiltration compared to Alpha challenged. Our data suggest that Delta has evolved to engage interferon responses in a manner that may enhance pathogenesis. The *in vivo* and *in silico* observations of this study underscore the need to conduct experiments with VOC strains to best model COVID-19 when evaluating therapeutics and vaccines.

## Introduction

The COVID-19 pandemic is being perpetuated by the emergence of “Variants of Concern” (VOC): mutant strains of SARS-COV-2 with enhanced disease-causing abilities. Early in the pandemic, strains obtained the D614G mutation that enhanced binding of the spike protein to the host hACE2 receptor. The mutation quickly became dominant among circulating strains around the world. Several potential variant strains were noted and monitored but few demonstrated any unique or enhanced differences in virulence or transmission. In September 2020, the B.1.1.7 strain was first identified in the United Kingdom and quickly spread to become the dominant variant worldwide in early 2021 [1]. Later, in December 2020, two other variants, Beta (B.1.351) and Gamma (P.1) were identified in South Africa and Japan/Brazil, respectively [2–4]. Both Beta and Gamma variants increased concerns because they decreased the efficacy of several vaccines in clinical trials [5–10].

In addition to the effects on vaccine efficacy in clinical trials, there were also reports that Alpha and Beta variants were able to cause enhanced disease in humans [11–16]. Animal studies with transgenic K18-hACE2-mice and hamsters supported those observations [17, 18]. Before circulating VOC established their dominance, two doses of Pfizer-BioNTech’s mRNA vaccine, BNT162b2, exhibited 95% efficacy in protecting individuals from severe COVID-19 in retrospective cohort studies of COVID-19-associated hospitalizations in early 2021 [19]. However, the efficacy of the BNT162b2 vaccine was slightly diminished in the context of Alpha (93.7% efficacy) [20].

As Alpha, Beta, and Gamma continued to spread, the Delta variant (B.1.617.2) was identified in India, preluding a massive surge of COVID-19 cases in the country [21]. Unsurprisingly, Delta was able to cause breakthrough cases of infection within a portion of fully vaccinated people, decreasing the overall efficacy of ChAdOx1, mRNA-1273, or BNT162b2 vaccines [20, 22–24]. In March 2021, the Delta variant was detected in the United States, and reports of its increased transmissibility necessitated investigation of its threat to both unvaccinated and vaccinated populations. Key pathogenic mutations in the B.1.617 SARS-CoV-2 lineage, which includes the Delta (B.1.617.2) and Kappa (B.1.617.1) VOC, occur within the spike protein, which mediates viral attachment and entry into host cells via the ACE2 receptor [25, 26]. These mutations have been reported to increase transmissibility among the population and decrease antibody neutralization [27, 28]. Delta does not harbor the N501Y substitution in the spike protein that was characteristic of the Alpha and Beta lineages but Delta does harbor the D614G substitution in spike, which contributes to the increased fitness and transmissibility of many VOC strains [29, 30]. Some strains of Delta are reported to harbor the K417N mutation previously found in Beta and Gamma, which sparked debate over designating the strain as a new variant [31, 32]. This mutant strain was labeled as “Delta Plus.” Additional mutations within the spike protein of Delta include P681R, which diverge from other VOC and help to enhance ACE2 receptor binding and cellular entry, contributing more to Delta’s pathogenicity [33].

To characterize the pathogenicity of Delta (utilizing strain B.1.617.2) and how it diverges from prior VOC (WA-1, Alpha, and Beta strains), we performed a time-course challenge study in K18-hACE2 transgenic mice which were intranasally challenged with Alpha and Delta strains. Through analysis of viral RNA load, tissue pathology, cytokine profiling, and total transcriptomics of the lung, we discovered indications that Delta causes increased interferon type I and II responses corresponding with greater lung inflammation when compared to Alpha. These data help to advance pre-clinical models of COVID-19 as well as serve as a benchmark to compare past and future VOC strains.

## Results

### The Delta variant of SARS-CoV-2 has a greater LD100 than Alpha and Beta VOCs in K18-hACE2 mice

**Fig 1:**
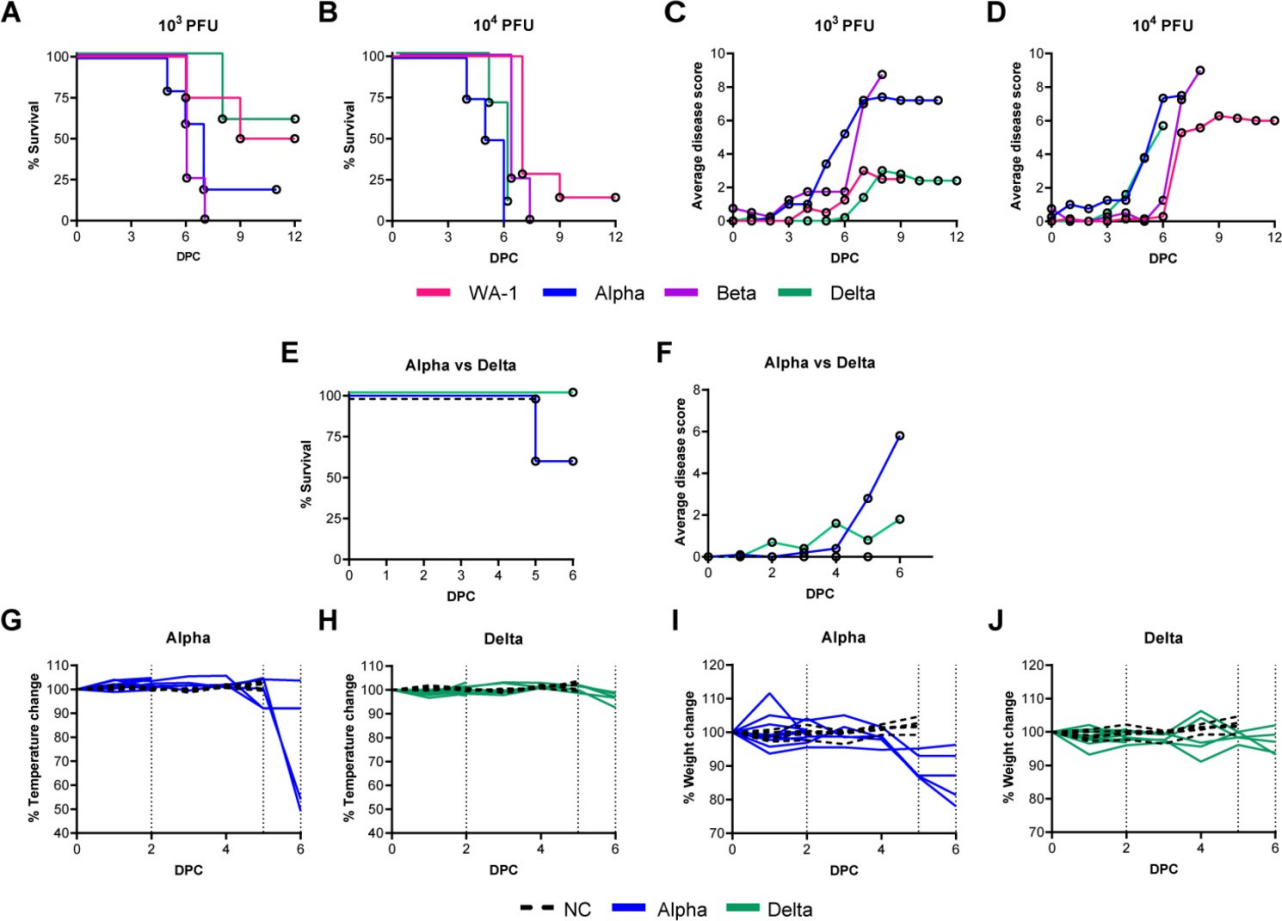
Survival, disease scores, weight, and temperature of K19-hACE2-mice challenged with WA-1, Alpha, Beta, and Delta strains. K18-hACE2 transgenic mice were intranasally challenged with 10^3^ or 10^4^ PFU WA-1, Alpha (B.1.1.7), Beta (B.1.351) or Delta (B.1.617) SARS-CoV-2, or mock-challenged with PBS (NC). Post-challenge, mouse survival (A, B) (n = 4 for 10^3^ WA-1, n = 7 for 10^4^ WA-1, n = 3 for 10^3^ Alpha, n = 4 for 10^4^ Alpha, n = 4 for 10^3^ Beta, n = 4 for 10^4^ Beta, n = 5 for 10^3^ Delta and n = 10 for 10^4^ Delta) and average disease scores per group on each day (C, D) were evaluated. Based on experimental results, mice challenged with Alpha (n = 10 split between timepoints) and Delta (n = 10 split between timepoints) or mock-challenged with PBS (n = 5), were evaluated at day 2 and day 6 post-challenge to measure survival (E), average disease scores (F), changes in rectal temperature (G, H) and bodyweight(I, J). Dotted lines indicate euthanasia points on day 2, day 5 (euthanasia of mock-challenge controls) and day 6. NC = no challenge.

To date, most K18-hACE2-mouse or Golden Syrian hamster studies have utilized ancestral viral strains of SARS-CoV-2 (e.g., WA-1, D614G, B.1 Wuhan), and few studies have been performed with emergent VOC [2, 34–38]. Early studies in K18-hACE-mice with a variety of viral strains used a wide range of challenge doses from as low as 100 PFU to as high as 10^5^ PFU [39–41]. In pilot studies with the Alpha and Beta variants, we evaluated 10^5^ PFU (high dose) and observed mortality as early as day 4 post challenge, suggesting and confirming that VOCs have enhanced virulence in K18-hACE-mice compared to WA-1 ancestral strain [42]. We then aimed to identify an appropriate challenge dose for WA-1, Alpha, Beta, and Delta VOC that could cause symptomatic disease for appropriate comparisons between the subtle aspects of variant pathogenicity that can be masked by lethal challenge doses. At the low dose of 10^3^ PFU, WA-1 challenge only resulted in 50% mortality of the mice (Fig 1A). We observed that a challenge dose of 10^3^ PFU using Alpha or Beta resulted in 80% and 100% mortality, respectively by day 7 post challenge. Although this comparison between Alpha and Beta challenge at 10^3^ PFU is not statistically significant (*P =* 0.6452), there is a significant difference between the survival of WA-1 challenged mice compared to Beta at the 10^3^ PFU dose, where WA-1 elicits higher survival (*P =* 0.0446). Despite the observations of enhanced virulence of Delta in humans, 10^3^ PFU challenge of K18-hACE2-mice mirrored WA-1 survival more so than Alpha or Beta (Fig 1A). As expected, increasing the challenge dose to 10^4^ PFU decreased survival of K18-hACE2-mice for all strains (Fig 1B). At the 10^4^ PFU challenge dose, Delta resulted in 0% survival (Delta vs WA-1 *P* = 0.0002) (Fig 1A and 1B). The 10^4^ PFU challenge dose also shortened the time to mortality for Alpha (WA-1 vs Alpha *P* = 0.0008). As we suspected from an ancestral strain with reduced virulence compared to emerging variants of concern, total mortality of WA-1 infected groups was not observed at either dose. Using a disease scoring system established previously [42, 43] we observed higher average disease scores for Alpha and Beta than Delta in K18-hACE2-mice intranasally challenged with 10^3^ PFU (Alpha vs Delta *P* = 0.0355; Beta vs Delta *P* = 0.0039) (Fig 1C and 1D). Interestingly, Delta’s observable disease phenotypes remained low by day 6 when Alpha- and Beta- challenged mice started to develop greater observable disease phenotypes consistent with morbidity (Alpha vs Delta *P* = 0.0056). Delta’s disease phenotypes were scored higher following 10^4^ PFU challenge, where they progressed in a pattern that was similar to Alpha. Delta-challenged mice received higher average disease scores than Beta by day 6 post challenge (Fig 1D). These experiments suggested that Delta compared to Alpha, and Beta causes mild disease in the K18-hACE2-mouse model (at 10^3^ PFU dose) despite its ability to cause severe disease in humans. We reasoned that these differences warranted further investigation.

### Mice challenged with of Alpha or Delta experience unique disease manifestation

To gain additional insights into the differences in pathogenesis of Alpha and Delta in K18 mice, we utilized a challenge dose of 10^3^ PFU which allows for differential disease manifestation between Alpha and Delta. Rectal temperature and weight loss were monitored in mice challenged with 10^3^ PFU of Alpha or Delta. In agreement with their lack of mortality and low disease scores (Fig 1E and 1F), Alpha-challenged mice had significant loss of body temperature (hypothermia) and weight loss between days 5 and 6 post challenge (Fig 1G and 1H). However, Delta-challenged mice maintained body temperature and did not experience weight loss (Fig 1I and 1J). At day 6 post-challenge, Alpha challenged mice had reached morbidity based on disease scoring and required euthanasia as a humane endpoint. However, at day 6 Delta challenged mice remained below euthanasia criteria. Collectively, these data demonstrate that Alpha and Delta cause distinct disease profiles in K18-hACE2-mice.

### Mice challenged with Delta have similar levels of viral RNA burden in the nares, and lungs but lower amounts of viral RNA in the brain

**Fig 2:**
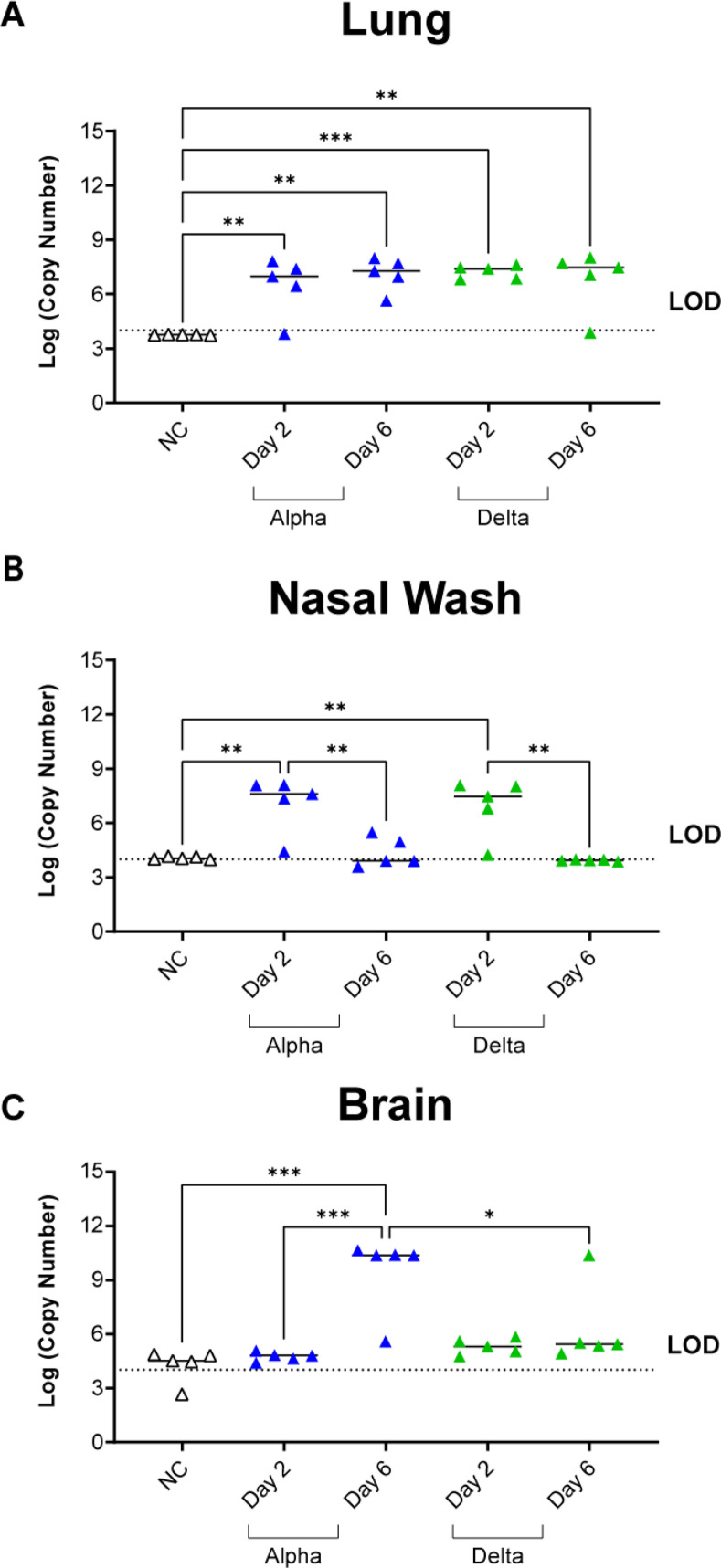
Viral RNA burden of disease-associated tissues was quantified using nucleocapsid qPCR. Viral RNA was detectable in the lung tissue of challenged mice from both variants at both timepoints compared to no challenge (One-Way ANOVA, left to right: *P =* 0.0082, 0.0011, 0.0008, 0.0029) (A). In Nasal Wash, viral RNA burden increased from both variants at Day 2 compared to no challenge (One-Way ANOVA, *P =* 0.0016 Alpha; *P =* 0.0030 Delta) and was decreased at Day 6 for both variants from copy numbers at Day 2 (One-Way ANOVA, *P* = 0.0043 Alpha; *P =* 0.0019 Delta) with no statistical difference between variants at each time point (B). Alpha-challenged mice have increased detectable viral RNA in the brain at Day 6 compared to Day 2 (One-Way ANOVA, *P =* 0.0005) while Delta challenge results in no viral RNA burden in the brain (One-Way ANOVA: *P =* 0.0002 NC vs Alpha Day 6, *P =* 0.0228 Alpha Day 6 vs Delta Day 6) (C). Dashed lines indicate limit of detection (LOD) via qPCR. NC = no challenge. Data is representative of n = 5 mice per experimental group.

It has been previously reported that SARS-CoV-2 viral burden is highest two days after challenge in mice [44], and based on our data we recognize that the Alpha variant will cause mortality at day 6 post-challenge; however, the Delta variant challenge did not cause the same disease, or survival (Fig 1A and 1B). Therefore, we aimed to evaluate viral RNA burden at day 2 and 6 post challenge for Alpha- or Delta-challenged mice. At euthanasia on day 2 and 6 the lung, brain, and nasal wash fluid were collected from challenged mice to quantify viral RNA burden via nucleocapsid qRT-PCR. We observed that viral RNA burden was remarkably similar (not statistically significant) between Alpha and Delta in the lung at both time points (Fig 2A). In the nasal wash, viral load was also detectable at similar levels (not statistically significant) for both Alpha and Delta at day 2. In contrast to the lung, the viral RNA burden decreased to undetectable levels at day 6 for both VOC (Fig 2B). Surprisingly, challenge with Delta only resulted in one mouse with detectable viral RNA in the brain at day 6 (Fig 2C). In contrast, mice infected with Alpha exhibited low RNA levels at day 2 and a 2-fold increase at day 6, suggesting an increase in viral replication in the brain over time (Fig 2C). These observations suggest that Delta at a challenge dose of 10^3^ PFU replicates efficiently in the airway in a manner similar to Alpha but Delta appears to have lower brain localization previously observed in K18-hACE2-mice [41, 44–46].

### Mice challenged with Delta experience significant inflammation in the lung tissue

**Fig 3:**
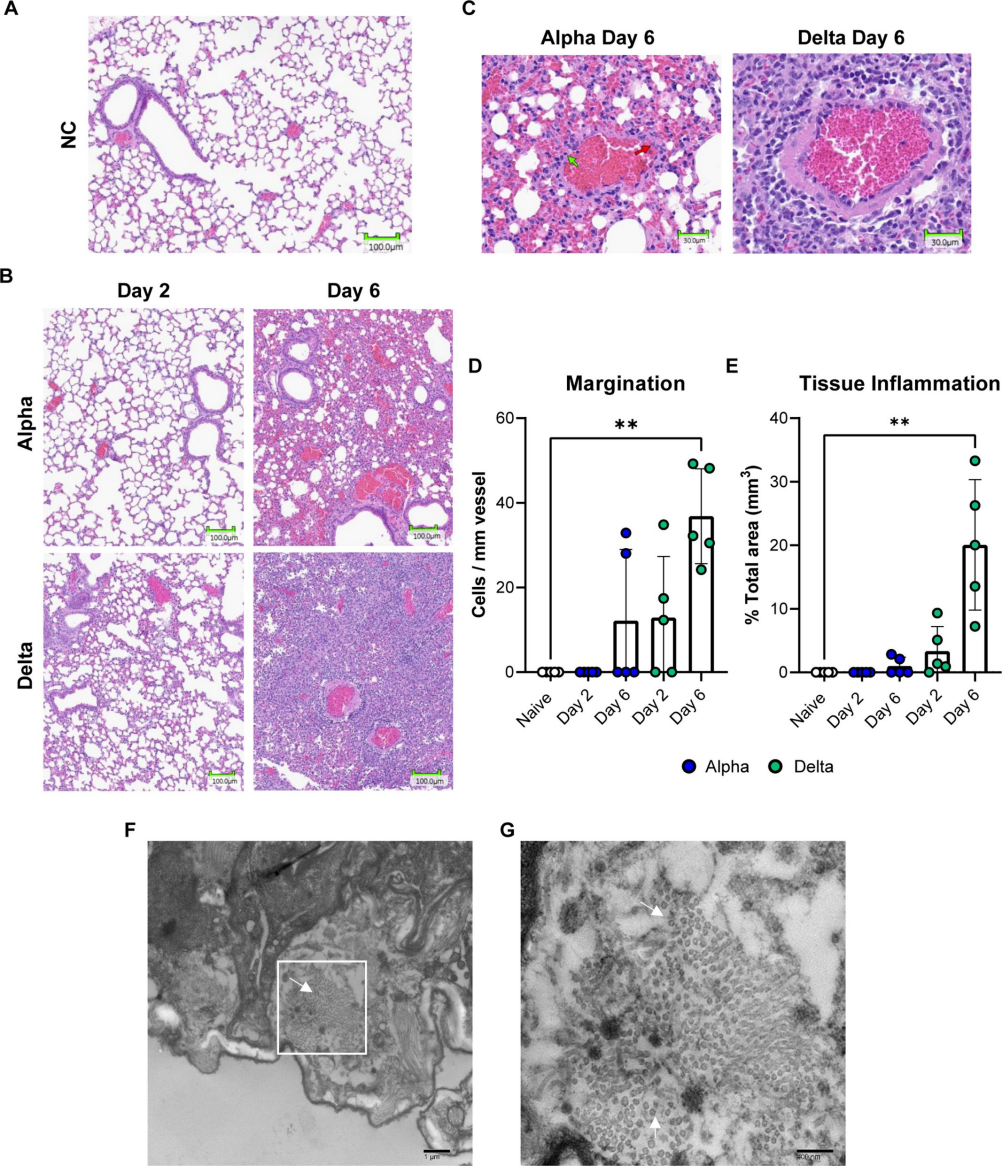
Histopathological and electron microscopy analysis of Alpha or Delta challenged K18-hACE2-mouse lungs. 20X images demonstrating inflammation in the lung tissue of Alpha and Delta SARS-CoV-2 challenged K18-hACE2 at Day 2 or 6 post-challenge (B) compared to no challenge (A). 20X images reveal margination at blood vessels within areas of inflammation (green arrows indicate counted marginating cells, red arrows indicate tissue-resident non-marginating cells)(C). Marginating inflammatory cells were counted within the tissue and were found to be increased in Delta challenge at Day 6 (Kruskal Wallis One-Way ANOVA, *P =* 0.0097) (D). Regional tissue inflammation was quantified as % total area of analyzed tissue (mm^3^) and was highest in Delta at Day 6 compared to no challenge (Kruskal Wallis One-Way ANOVA, *P* = 0.0012) (E). Error bars represent SD. Electron microscopy images displayed distribution of Delta virus particles in 1x10^3^ PFU challenged mouse lung tissue (F,G). White arrows identify virions. Cell counts were reported for n = 5 mice per experimental group.

Based on qRT-PCR data showing viral RNA burden in the lung but not brain of 10^3^ PFU Delta-challenged mice, we hypothesized that relative to Alpha, Delta may cause more robust pneumonia and less encephalitis in K18-hACE2-mice. To test this, we performed histopathological analysis on the lungs collected at days 2 and 6 to characterize the pneumonia caused by Alpha or Delta challenge. At two days following challenge, inflammation, and recruitment of inflammatory cells could already be observed in the Delta- but not Alpha-challenged mice when compared to the lungs of no challenge mice (Fig 3A and 3B). At this time, a pathologist identified that the inflammatory infiltrate was composed predominantly of lymphocytes and occasional histiocytes. By day 6, an increase in marked perivascular inflammation and margination was measured in Delta- (36.86 marginating lymphocytes per mm length of endothelium) and Alpha-challenged mice (12.19 marginating lymphocytes per mm length of endothelium) (unpaired t-test *P =* 0.026) (Fig 3C and 3D). In challenged Alpha lungs, less total vessels were identifiable within areas of inflammation, awarding these samples a margination score of zero cells/mm of vessel. Overall, less inflammation occurred in Alpha lungs. At day 6, only 1% of the lung area featured inflammation in Alpha, compared to 20% for Delta-challenged mice (unpaired t-test *P =* 0.003) (Fig 3E). Within these tissue areas (the airway, alveoli, and thin mucous layer), the presence of Delta virions was identified using electron microscopy (Fig 3F and 3G). Collectively, these data suggest Delta-challenged mice have increased cellular responses and inflammation compared to Alpha-challenged K18-hACE-mice.

### Pro-inflammatory cytokines are increased in mice challenged with Delta at day 6

**Fig 4:**
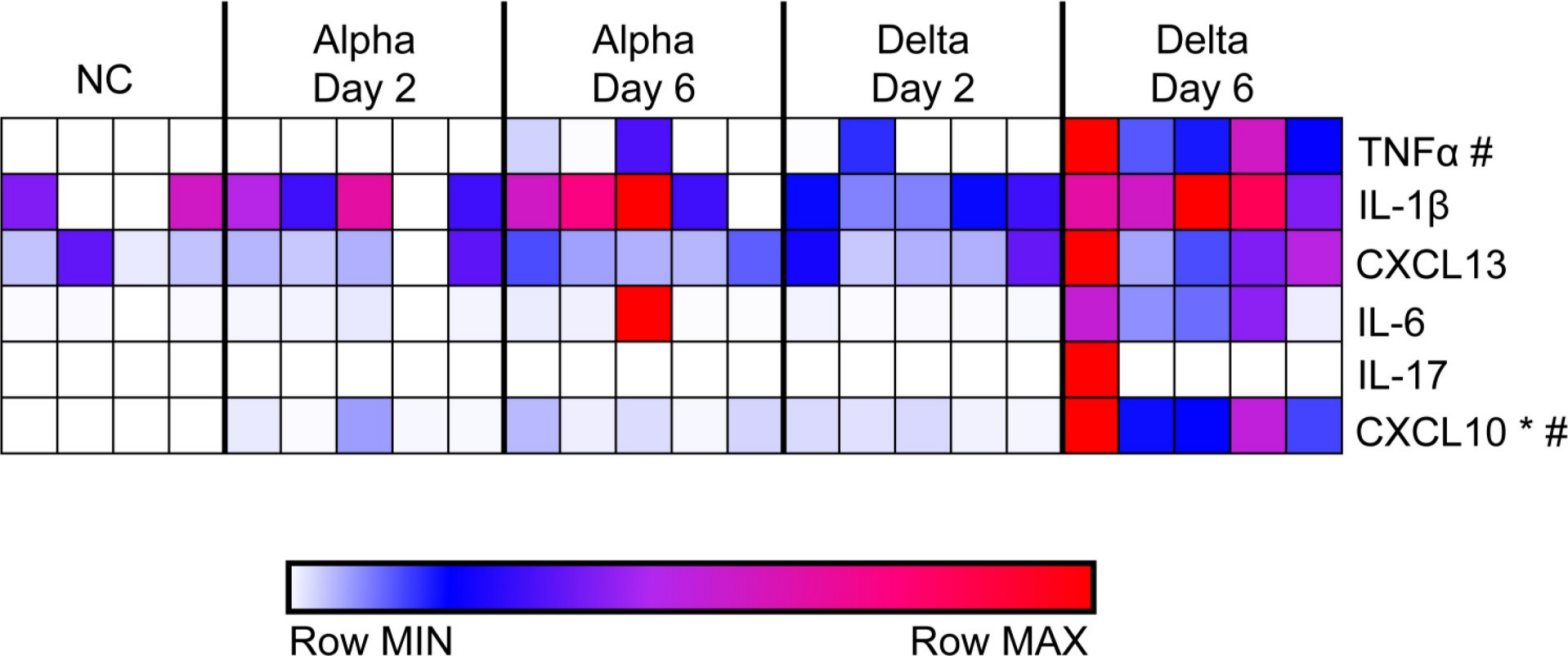
Analysis of cytokines production in lung supernatants from Alpha or Delta challenged K18-hACE2 transgenic mice at 2- or 6-days post-challenge. Non-challenged, Alpha- or Delta-challenged lung supernatants were used to determine local cytokine production in response to challenge. Concentrations (pg/mL) were graphed using Morpheus to reveal relative levels of cytokines compared to non-challenged lungs. * = statistical significance between Alpha day 6 and Delta day 6 (One-Way ANOVA *P =* 0.0105). # = statistical significance between no challenge and Delta Day 6 (One-Way ANOVA *P =* 0.0225 TNFα; *P =* 0.0058 CXCL10). (n = 5 except in NC where n = 4).

Histopathological analysis revealed significant inflammation in the lungs of Delta-challenged mice at day 6 compared to Alpha-challenged mice despite equal viral RNA burden. To better understand differences in the inflammatory profile of these tissues, pro-inflammatory cytokine levels in lung supernatant at day 2 or 6 were quantified to profile strain specific variations in the “cytokine storm” [47]. Insignificant amounts of cytokine were produced in the lungs of mice challenged with Alpha or Delta at day 2 (Fig 4). Alpha-challenged mice produced low amounts of IL-1β and CXCL13 compared to no challenge mice at day 2 or 6. By contrast, the cytokine response to Delta challenge at day 6 compared to no challenge revealed high levels of TNFα, and CXCL10 (One-Way ANOVA *P =* 0.0225, *P =* 0.0058) (Fig 4). CXCL10 was the only cytokine which statistical analysis proved to be higher in the lung supernatant of Delta-challenged mice than Alpha (One-Way ANOVA *P =* 0.0105). The inflammatory response within the lungs of mice challenged with Delta was elevated compared to Alpha. To gain insight into the specific mechanisms of the immunological host response, we performed RNAseq to characterize the total transcriptional profile of Alpha- and Delta-challenged K18-hACE2-mouse lungs.

### RNAseq analysis of lung tissues from mice challenged with Alpha or Delta shows no difference in viral sgRNA

**Fig 5:**
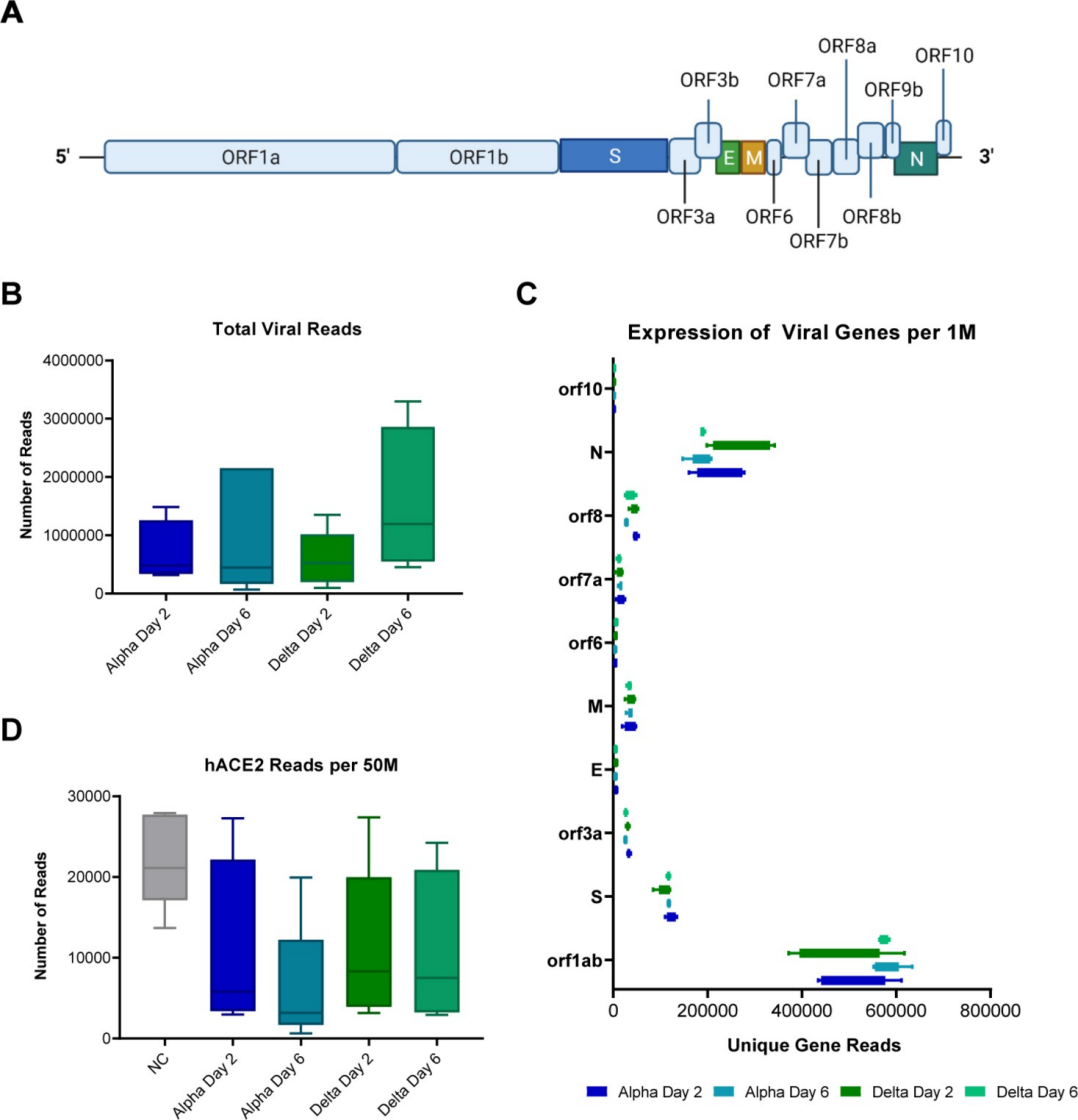
RNAseq analysis of SARS-CoV-2 genes in challenged mouse lungs. Schematic of the SARS-CoV-2 genome (A). Total viral read counts in each group (B) as well as quantification of viral reads per 1M RNAseq reads obtained (C). Reads of human ACE2 gene of the K18-hACE2-mice were quantified per 50M reads obtained per lung sample (D). (n = 4 in Alpha Day 2 and Delta Day 6; n = 5 in Alpha Day 6 and Delta Day 2).

We utilized RNA that was isolated from the lung tissue of challenged and no challenge mice at 2- and 6-days post-challenge for Illumina transcriptomic analysis. RNA sequencing reads were first mapped to the SARS-CoV-2 viral genome to quantify the expression levels of viral genes over the course of infection (Fig 5A). This revealed no significant difference between the two strains (Fig 5B and 5C) which directly supported the qRT-PCR analysis (Fig 2). It is known that SARS-CoV-2 infection results in down-regulation of hACE2 expression [48, 49]. Therefore, we mapped RNA reads to the hACE gene to analysis this effect. Although not significant, a trend of lowered hACE2 expression occurred in Alpha challenged mice, whereas Delta challenged mice appeared to experience only slightly reduced hACE2 expression (Fig 5D). These observations together with qRT-PCR nucleocapsid data described above, suggest that with equal viral burden (same challenge dose and equal viral RNA burden), the differences in LD100 of Alpha and Delta as well as the disease phenotypes are likely related to differences in host response to VOC and not overall viral load.

### Delta challenge causes a higher magnitude of host gene expression change compared to Alpha

**Fig 6:**
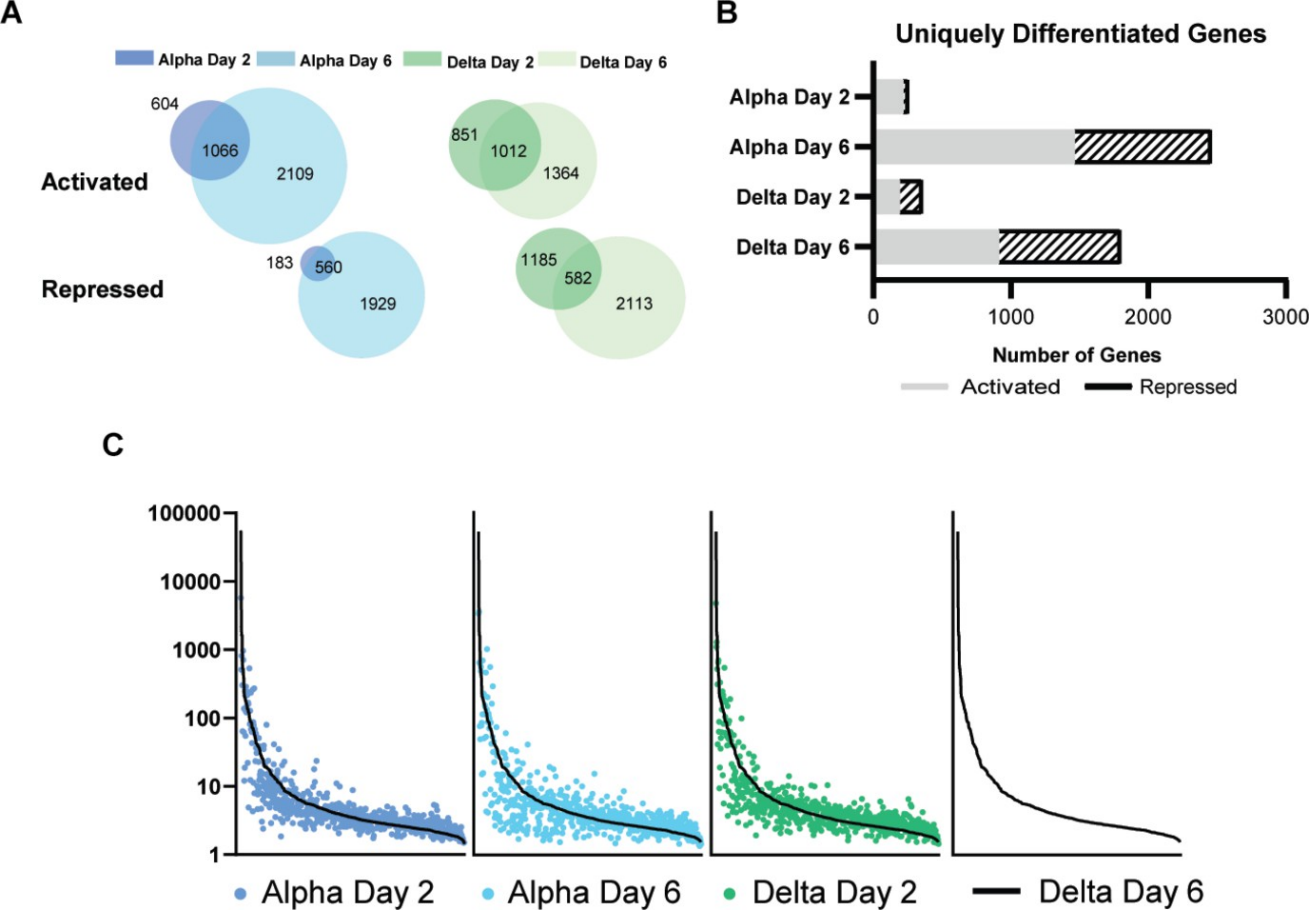
Total transcriptomic analysis of mouse genes in non-challenged mice compared to Alpha and Delta challenged mice at days 2 and 6. RNAseq reads from challenged mice were compared to non-challenged mice to determine differential gene expression (A). Genes that were statistically significant are indicated in relation to challenge day and their relative expression as being activated or repressed. Venn diagrams are shown to illustrate the amount of overlap in significant genes between days 2 and 6. The total number of unique differentially expressed genes are represented per each challenge day and relative expression (B). (n = 4 in Alpha Day 2 and Delta Day 6; n = 5 in Alpha Day 6 and Delta Day 2).

To compare the host responses of Delta- and Alpha-challenged mice, we mapped lung RNA sequencing reads to the mouse genome. We observed that Alpha or Delta challenge in K18-hACE2-mice result in distinct transcriptional changes. Two days after challenge with Alpha, 2,413 statistically significant genes (FDR *P* ≤ 0.04) were differentially expressed compared to no challenge mice, a difference which increases more than two-fold to 5,664 genes at day 6. In Delta groups, differential expression encompasses 3,048 and 4,489 genes respectively. Venn diagrams of the gene sets comparing by variant and time-points illustrate overlaps in gene sets that are either activated or repressed. Both activated and repressed gene sets increase from day 2 to 6 (Fig 6A). Further filtration of these sets of genes to identify those that are uniquely affected by VOC challenge and timepoint revealed that Alpha challenge results in more genes differentially regulated at day 6 than Delta challenge (Fig 6B). A set of core-activated genes (S2 Table) was identified through a four-way comparison of VOC and timepoint. Despite their different disease pathology, Alpha and Delta challenge activate a similar set of host genes with differences lying in the extent to which each gene is respectively regulated. Common genes which were activated by Alpha challenge at day 2 were also activated at day 6 and by Delta challenge at both times with different fold changes. Commonly repressed genes followed a similar trend. Together, these trends support that the nuances of the host response to Alpha and Delta infection may largely contribute to the differences that are seen in disease pathology.

### GO analysis revealed unique systems of enriched genes in mice challenged with Delta

**Fig 7:**
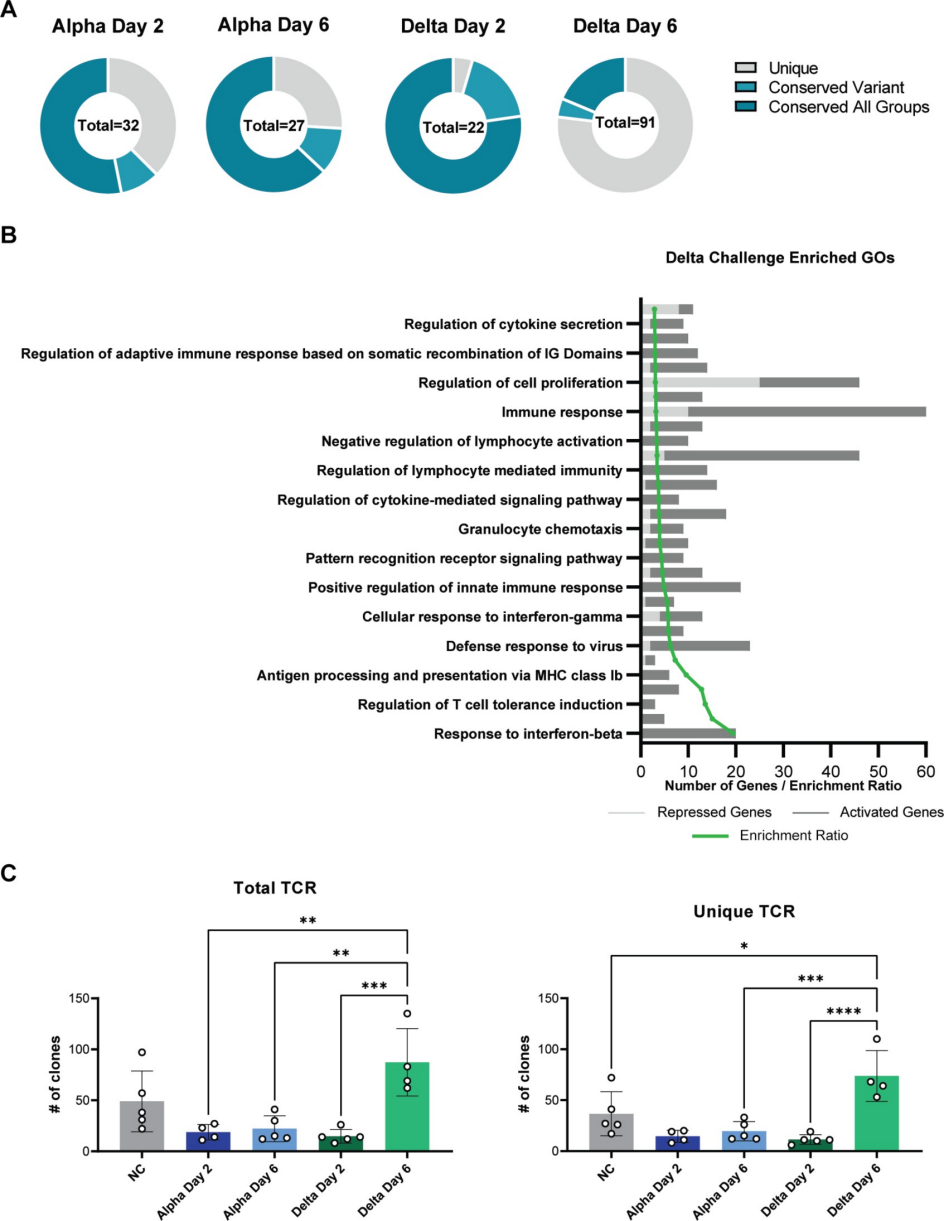
Gene ontology analysis of systems of genes in challenged mice. Circle plots indicate the number of unique or conserved sets of genes that were found to be enriched per each group (A). The top 30 enriched GO terms are shown and plotted per their relative level of expression and enrichment ratio (B). TCR clonotypes were mapped and represented as Total clones (C) or unique clones per unique CDR3 sequence (D). Total TCR clones (One-Way ANOVA *P* = 0.0005) and unique clones were increased between Delta day 2 and day 6 (One-Way ANOVA *P*<0.0001). (n = 4 in Alpha Day 2 and Delta Day 6; n = 5 in Alpha Day 6 and Delta Day 2).

Gene Ontology (GO) term analysis was performed to systematically group transcriptomic observations to identify specific pathways and gene systems whose expression changes in response to Alpha and Delta challenge. Relative to Alpha-challenged mice, Delta-challenged mice had a higher number of unique GO terms that were enriched at day 6 (Fig 7A). To categorize the specifically enriched terms in Delta at day 6, pathway enrichment ratios were calculated for the top 30 GO terms (Fig 7B). The expression of genes that were annotated with GO terms pertaining to immune responses, anti-viral, or lymphocyte recruitment were increased, as were genes of pathways associated with T cells and responses to IFN-β (Fig 7B). To deepen the finding by GO term analysis that T cell response genes are enriched in Delta challenged mice, we performed T cell receptor (TCR) clonotype analysis using RNAseq reads. Within the no challenge control mice, we detected ~50 total clonotypes. In Alpha-challenged mice, the number of clones were decreased relative to no challenge at both day 2 and 6. By contrast, Delta-challenged mice had decreased clones at day 2, but we observed a 6-fold increase in TCR clones at day 6 (Fig 7C). In counts of unique TCR clonotypes, the same trends were observed: at day 6, Delta challenge had the most total unique TCR clones compared to non-challenged mice, day 6 post Alpha-challenged mice, and day 2 post Delta-challenged mice (Fig 7D). These data suggest that Delta may induce more robust T cell response.

### Delta challenge causes increases interferon dependent gene expression

**Fig 8:**
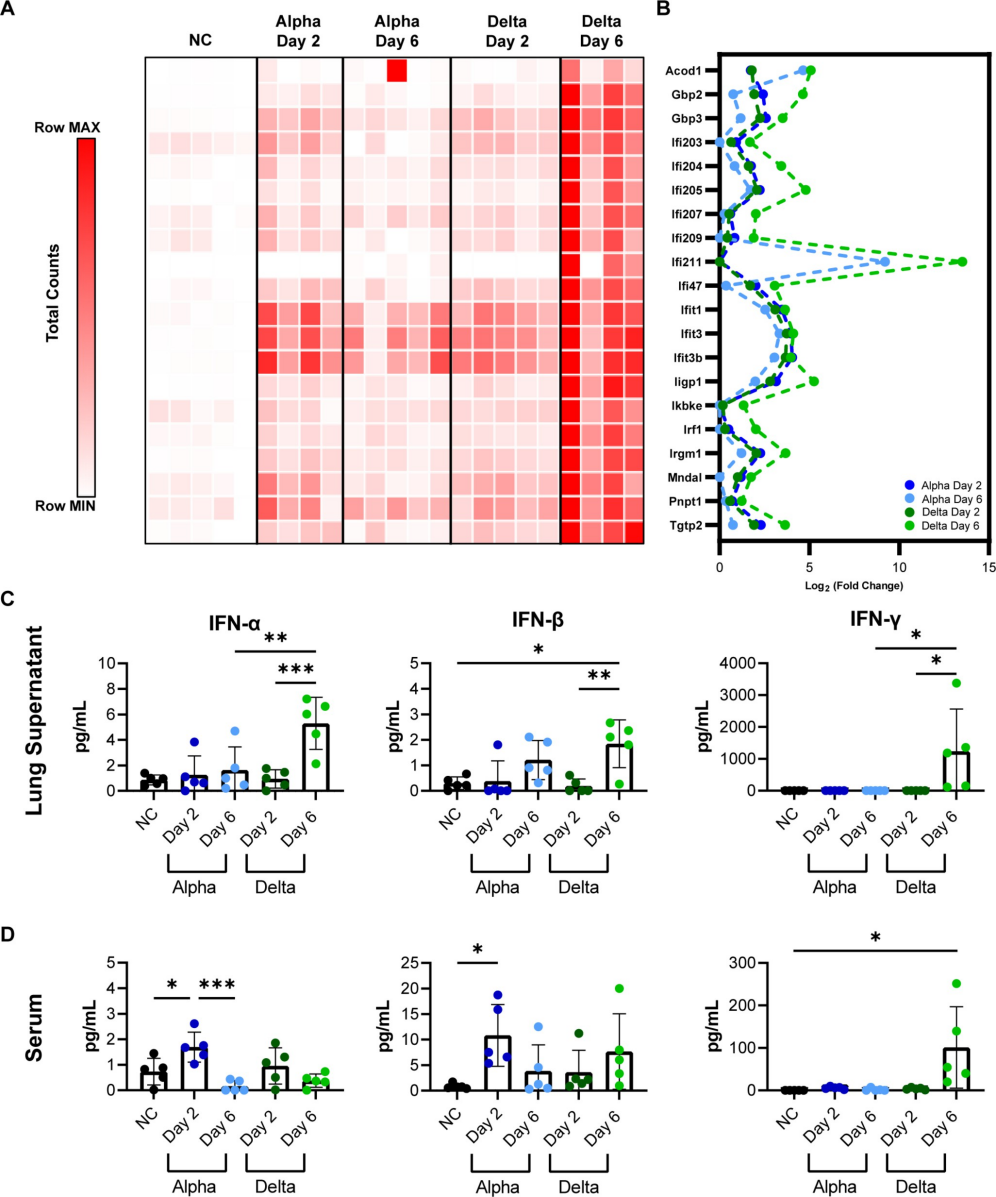
Analysis of interferon dependent gene expression and production of type I and type II interferon in lungs and serum of K18-hACE2-mice. Total RNA counts per interferon dependent gene are shown per each mouse, time-point, and variant challenge group (A). Each square represents the total RNA counts per gene from one mouse. Interferon dependent genes are shown with relative fold change plotted (B). Interferon alpha, beta, and gamma production in lung supernatant (C) and serum (D) of K18-hACE2-mice. (n = 5).

Gene expression analysis focused by GO terms revealed Delta challenge induced expression of interferon response genes (Fig 7). Interferon is an important regulator of the antiviral response in humans. It’s variable expression in response to specific VOC strains of SARS-CoV-2 exemplifies the intensity of differences in the host response. TGTP1 and IFI211, two interferon related genes, are the highest-expressed genes in Delta at day 6 at a fold change of 52,725 or 11,733 higher than in no challenge mice. In Alpha at day 6 these genes are 3,367 and 591-fold higher than no challenge. Due to the important role of interferons in the anti-viral response to SARS-CoV-2 which may be differentially engaged by VOC, we continued to compare the top 20 genes involved in general interferon responses. Total gene counts in lung RNA from each experimental group are shown in Fig 8A. Delta at day 6 shows high interferon dependent gene expression as well as the highest fold change difference (Fig 8B). Collectively, these data suggest Delta challenge results in higher interferon response. To test that hypothesis, we directly measured type I and II interferons in lung supernatants and serum. Modest production of IFN-α or IFN-β were observed in both lung and serum by both variants (IFN-α: *P* = 0.0008 Delta Day 6 vs No Challenge in lung supernatant; *P = 0*.*0456* Alpha Day 2 vs No Challenge in serum; IFN-β: *P =* 0.0126 Delta Day 6 vs No Challenge in Lung Supernatant; *P* = 0.0474 Alpha Day 2 vs No Challenge in serum); however, Delta challenge resulted in higher IFN-γ at day 6 compared to day 2 and both Alpha challenge timepoints (*P* = 0.0265 in lung supernatant, *P* = 0.0103 in serum compared to no challenge) (Fig 8C and 8D). This dynamic production of interferon subtypes that appears to be unique to challenge strain and timepoint, speaks to the interesting differences in host response that may set apart variants in the K18-hACE2 SARS-CoV-2 challenge model. As variants continue to appear and be incorporated into current research projects, these phenotypic and pathological changes will need to be appreciated.

## Discussion

In this study, we modeled the pathogenesis of SARS-CoV-2 variants of concern in the K18-hACE2-mouse challenge model. Based on our observations that the Delta variant differs in disease manifestation from Alpha and Beta VOC-challenge, we performed a transcriptomic experiment to compare Alpha- and Delta-challenged mice at disease-significant timepoints. Despite a similar viral RNA burden in the respiratory tract at day 2 and 6, Delta causes more significant inflammation in the lungs than Alpha as infection progresses. The host response also varies over time, with Delta causing increased antiviral cytokine production, specifically IFN-γ, at day 6. GO term analysis of Delta-challenged mice suggested a greater number of immunological pathways that are implicated at time points in the variant’s pathogenesis compared to Alpha, further indicating the unique nature of the host response that is mounted against each individual variant of SARS-CoV-2.

**Fig 9:**
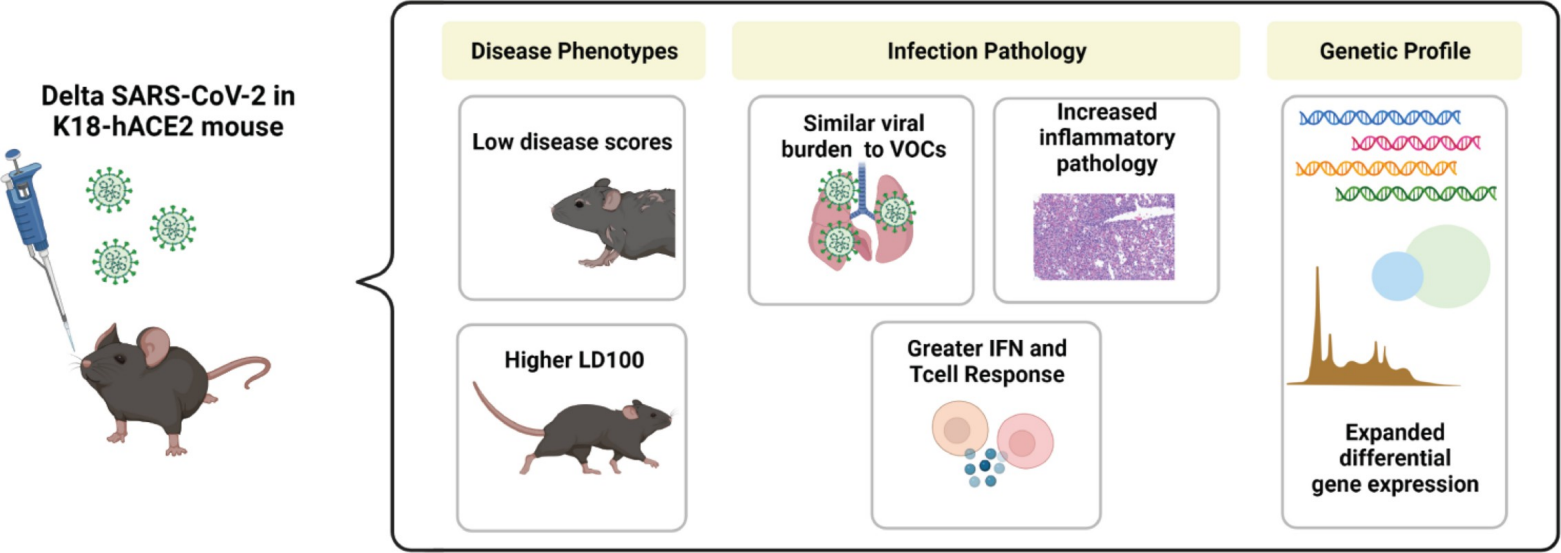
SARS-CoV-2 Delta variant challenge in the K18-hACE2 transgenic mouse model. Male, 8 week-old K18-hACE2 transgenic mice were intranasally challenged with 10^3^ PFU Delta SARS-CoV-2 and the host response to virus was analyzed at 2 and 6 days post-challenge. Delta challenge results in attenuated disease scores compared to previous VOC, and extended survival time at this dose. Despite similar viral RNA burdens, Delta-challenged mice experience greater inflammatory pathology in the lung with an expanded differential gene expression profile suggestive or greater interferon and T cell responses.

Severe COVID-19 is perpetuated by the dysregulated production of pro-inflammatory cytokines, including type I and II interferons, in a “cytokine storm” pathology [47, 50, 51]. We observed that CXCL10, a chemokine that is produced downstream of IFN-γ signaling, in addition to IFN-α, IFN-β and IFN-γ were significantly higher in the lung supernatant of mice 6 days after Delta variant challenge, than in mice after Alpha challenge. RNAseq analysis corroborated these findings, with higher expression of interferon pathways genes in the lung tissue. The interferon signaling pathways, and the increased expression of interferon-stimulated genes (ISG) in COVID-19 patient lung tissues implicate the critical role of interferon in SARS-CoV-2 pathogenesis, but it has not been determined if the cytokines contribute more to suppressing viral replication, or augmenting the immune response through their dual pro- or anti-inflammatory abilities, thus contributing to greater disease [50, 51]. Delta challenge causes a significant increase in lung and serum IFNγ which is greater than what has been previously reported in K18-hACE2-mice using different challenge strains such as ancestral WA-1 [41, 44] IFNγ is a potent antiviral cytokine that is produced later in the timeline of viral challenge and often may not be observable in widely used mouse models of lethal SARS-CoV-2 challenge because of their short survival timelines [52]. IFNγ production is typically associated with the activation of natural killer cells, innate lymphoid cells, or Th1-like adaptive immune cells [45]. Its high concentrations in the lung of Delta mice could corroborate greater recruitment of these cell populations identified in histopathology data as infiltrating cells (Fig 3). Together the observations of this study suggest that Delta challenge causes enhanced innate immune responses compared to Alpha (Fig 9). Liu et al. in their comparison of Delta and an ancestral strain, examined cell infiltration by flow cytometry and immunofluorescence staining and confoundingly reported fewer inflammatory cells in Delta lungs than ancestral-strain despite very similar cytokine profiles between variants [53]. While our results alone only begin to suggest a differential engagement of interferons by Delta during its host pathogenesis, and combined with Liu et al.’s data show an abrogated cellular response between Delta and prior VOC strains, further comprehensive work would be necessary to fully map out the timelines of signaling mechanisms and cellular response profiles responsible for unique VOC pathology.

Surprisingly, we observed that the viral RNA burden of the respiratory tract was identical per day and per variant in the K18-hACE2-mice, as determined by both RT-qPCR and RNAseq. A clear question then remains: why does Delta induce a greater local inflammatory response in the lung? One study from Stolp, et al. demonstrated that SARS-CoV-2 variants differentially reduce the numbers of interferon-producing immune cells in K18-hACE2-mouse tissues, although it lacked the inclusion of Delta in its comparison of Alpha, Beta, Gamma, and ancestral strains [54]. Another possible mechanism that could be explored is the interaction of viral sgRNAs in regulating host gene expression. Open Reading Frames (ORFs) of SARS-CoV-2 have been implicated in controlling the host anti-viral response [55]. Studies report that the increase in viral burden due to Alpha leads to increases in some but not all sub-genomic RNAs from the genomic regions harboring variant-characteristic mutations [56]. Another study links the expression of sub-genomic RNAs in Alpha infection, specifically ORF9b, to antagonism of the host immune response and suppression of ISGs which would allow for more efficient viral replication and enhanced transmission [57]. Although viral gene reads in our analysis show no significant differences between Alpha and Delta sgRNAs, it is possible that our selected timepoints were too late to detect the differences previously reported at 10 and 24 hours post challenge, or that our challenge dose was too low. Still, our abundant transcriptomic data suggest an extensive list of gene pathways that were enriched during Delta but not Alpha infection.

We observed that 10^3^ PFU challenge with Delta variant does not cause the dramatic weight loss and hypothermia reported in models of Alpha and Beta challenge [17]. One well known caveat of the K18-hACE2-mouse model is the fact that WA-1, Alpha and Beta strains enter the brain [44, 46, 58–64]. However, it appears that the Delta variant remains more localized in the lungs of challenged mice (Fig 3). At higher challenge doses (10^4^ PFU), our lab and others have shown that viral RNA can be detectable in the brain as early as day 6 following Delta challenge [16, 43]. In data not yet published, we have also observed that at later time points, 10^3^ PFU challenge with Delta can still result in some brain tissue burden, based on qRT-PCR analysis. It’s generally accepted that K18-hACE2-mice reach mortality due to brain infection, supported by manifestations of severe disease including hypothermia, and heightened inflammatory profiles. Seehusen et al. showed that Delta’s infectious potential in neurons is equal to that of previous VOC [65]. It is possible that a delay in the entry of Delta to the murine brain allows for an increased survival time and leaves more time for the development of severe infection and pneumonia in the lung. However, viral kinetics studies would be required to further measure this.

We appreciate that the K18-hACE2-mouse model has caveats to consider. The transgenic model expressing human ACE2 in addition to mouse ACE2 addresses SARS-CoV-2’s higher affinity for binding human ACE2, which reduces species tropism and allows virus to cause lethal disease in the mice. As is common in transgenic protein expression, hACE2 under the epithelial cell cytokeratin-18 (K18) promoter is not expressed with the identical tissue localization as it would be in humans. Therefore, viral localization should always be carefully considered. Our observations show that Delta accomplishes lung infection in the mouse and induces strong pneumonia. One popular alternative to the K18-hACE2-mouse model is the Syrian Golden Hamster. After SARS-CoV-2 challenge, hamsters develop more observable respiratory disease and pneumonia due to a higher affinity for hamster ACE2 [66, 67]. Delta has been reported to be highly pathogenic in golden hamsters, with increased tropism for the respiratory tract and low development of disease phenotypes like weight loss and drops in temperature [33, 68]. The similarities between mouse and hamster models provide evidence for Delta’s unique disease manifestation, defending the importance of its use in preclinical models when compared to prior strains.

With the emergence of the Omicron variant, it’s clear that novel variants of SARS-CoV-2 will continue to arise with enhanced disease capabilities. To continue preclinical COVID-19 research with small animal models, strains that more accurately recapitulate present human disease are necessary. Understanding the unique disease mechanisms of each VOC and the caveats they may present in these models will help to advance research into the identification or improvement of therapeutics.

The data presented here show that a sublethal challenge with Delta induces inflammation that is more histopathological than the Alpha VOC and increases interferon responses. This study also defines the doses of SARS-CoV-2 VOC that are useful for considering in performing preclinical studies. Our findings of enhanced pathogenesis from the Delta variant compared to the Alpha variant recapitulate some of the findings of Liu, et al. where the Delta variant at a higher challenge dose was found to engage the murine host immune response differently from an ancestral SARS-CoV-2 strain [16]. The use of RNAseq allowed us to characterize the entire transcriptome of the mouse lung to further understand the effect of challenge by either Alpha or Delta variants. We observed massive differential gene expression caused by Delta despite that fact that the mice did not reach mortality. Our study identified unexpected and uncharacteristic aspects of Delta’s disease pathogenesis. Together, our study outcomes underscore the need to continue to understand SARS-CoV-2 especially outside of the immunogenicity differences of the spike protein that are most often focused upon. We plan to build upon the findings of this study and characterize single-cell gene expression of the host response in a continued effort seeking to understand ways to combat this virus.

## Materials and methods

### Biosafety, animal, and ethics statement

K18-hACE2-mice purchased from Jackson Laboratory (B6.Cg-Tg(K18-ACE2)2Prlmn/J; JAX strain number #034860) were used for *in vivo* challenge studies under the approval of West Virginia University IACUC protocol number 2009036460. SARS-CoV-2 viral propagation work in addition to the challenge studies were conducted in West Virginia University’s High Containment Biosafety Laboratory Level 3 facility under the IBC protocol number 20-04-01. Tissue samples including SARS-CoV-2-challenged mouse serum and lung supernatant collected in BSL3 experiments were treated with 1% Triton (per volume, TRI Reagent (Zymo R2050-1), or formalin-fixedbefore exiting high containment for use in BSL2.

### SARS-CoV-2 cultivation and K18-hACE2 mouse challenge

WA-1, Alpha, and Beta strains of SARS-CoV-2 were obtained from BEI: USA-WA-1/2020 (WA-1; BEI NR-52281) (GenBank accession number: MN985325), hCoV19/England/204820464/2020 (Alpha; NR-54000)(GISAID: EPI_ISL_683466), and hCoV19/South Africa/KRISP-EC-K005321/2020 (Beta; BEI NR-54008) (GISAID: EPI_ISL_678570). The SARS-CoV-2 Delta variant (B.1.617.2 hCoV-19/USA/WV-WVU-WV118685/2021) was obtained from a patient viral transport medium swab (GISAID Accession ID: EPI_ISL_1742834). All SARS-CoV-2 strains were propagated in Vero E6 cells (ATCC-CRL-1586) to prepare viral stocks. All viral strains were NGS sequenced to confirm the strains’ genomes. No mutations were identified that would suggest altered pathogenesis. For variant comparison studies, K18-hACE2 mice were anesthetized with an IP injection of ketamine (Patterson Veterinary 07-803-6637) /xylazine (Patterson Veterinary 07-808-1947) then intranasally challenged with a volume of 50 uL (25uL per nare) at: 10^3^ PFU/dose (8 week old male and female received WA-1 or Beta; 12 week old female mice received Alpha; 13 week old female received Delta), or 10^4^ PFU/dose (17 week old female received WA-1; 8 week old male and female received Alpha or Beta; 20 week old female received Delta). Mouse cohort details are summarized in S1 Table. For timepoint comparison study, male age-matched mice at 8 weeks old were infected with 10^3^ PFU. Viral doses were prepared from the first passage collections from infected Vero E6 cells.

### Disease score of SARS-CoV-2 challenged mice

K18-hACE2 mice were evaluated daily after viral challenge using in-person health assessments in the BSL3 as well as the SwifTAG Systems video monitoring until the planned euthanasia timepoint. Health assessments of the mice were scored based on the following criteria: weight loss (, appearance, activity, eye closure, and respiration using a scaling system that has been described previously [42, 43]. Each day, mice were awarded an additive score comprised of the five sub-scores. Mice that scored 5 or above on the health assessment or reached 20% weight loss required immediate euthanasia. For reporting purposes, the scores of each mouse in a group were combined each day and reported as the average.

### Euthanasia and tissue collection

Mice were euthanized using an IP injection of Euthasol (390mg/kg) (Pentobarbital) followed by cardiac puncture. The blood from cardiac puncture was collected in BD Microtainer gold serum separator tubes (Catalog No: 365967), and spun down at 15,000 x *g* for 5 minutes to separate the serum. Nasal wash was performed by pushing 1mL of PBS through the nasal pharynx and collected in a microcentrifuge tube. 500μL of nasal wash was added to 500μL of TRI reagent for RNA purification and the remainder of the nasal wash was frozen and saved for future serological work. Lungs were dissected and separated: the right lobe of the lung was homogenized in 1mL of PBS in gentleMACS C tubes (order number: 130-096-334) using the m_lung_02 program on the gentleMACS Dissociator. For RNA purification, 300μL of lung homogenate was added to 1000μL of TRI Reagent (Zymo research) an additional 300 μL of lung homogenate was centrifuged at 15,000 x *g* for 5 minutes to collect the lung supernatant for cytokine analysis. The left lung was fixed using 10% neutral buffered formalin for histopathology. Whole brain tissue was also homogenized in 1mL PBS in gentleMACS C tubes using the same setting as lung on the gentleMACS Dissociator. For RNA purification from brain, 1000μL of TRI Reagent was added to 500μL of brain homogenate.

### qPCR SARS-CoV-2 viral copy number analysis of lung, brain, and nasal wash

The Direct-zol RNA miniprep kit (Zymo Research R2053) was used to purify RNA from lung, brain, and nasal wash following the manufacturer’s protocol. SARS-CoV-2 copy numbers were assessed through qPCR using the Applied Biosystems TaqMan RNA to CT One Step Kit (Ref: 4392938) and a previously described reaction utilizing the nucleocapsid primers (F: ATGCTGCAATCGTGCTACAA; R: GACTGCCGCCTCTGCTC); and TaqMan probe (IDT:/56-FAM/TCAAGGAAC/ZEN/AACATTGCCAA/3IABkFQ/) synthesized according to Winkler. *et al*., 2020 [43, 44]. Purified RNA samples with a concentration less than 100ng/uL were not diluted for use in qPCR reactions. All Nasal Wash RNA samples were used at a set volume of 2 uL due to low RNA quantification Each sample reaction was prepared in triplicate and analyzed in MicroAmp Fast optical 96 well reaction plates (Applied Biosystems 4306737) on the StepOnePlus Real-Time System machine using the following parameters: Reverse transcription for 15 minutes at 48°C, activation of AmpliTaq Gold DNA polymerase for 10 minutes at 95°C, and 50 cycles of denaturing for 15 seconds at 95°C and annealing at 60°C for 1 minute.

### Luminex and Meso Scale Discovery cytokine analysis

The R&D 5-plex mouse magnetic Luminex assay (Ref LXSAMSM) was used to quantify cytokines: IL-1β, CXCL13, TNFα, IL-6, IFN-γ, IL-17, and CXCL10 in lung supernatant. Manufacturer protocols were followed to prepare samples. Mouse cytokine plate was analyzed on the Luminex Magpix and concentration values (pg/mL) were calculated based off standard curves generated for each cytokine in the assay. IFN-α, IFN-β and IFN-γ were additionally quantified using MSD U-PLEX Interferon Combo 1 (ms) assay kit (Catalog No K15320K-1) and manufacturer protocols. MSD assay plates were analyzed using the Meso Scale Discovery Sector 2400.

### Histology and electron microscopy

The left lobes of lungs were immediately fixed in 10mL of 10% neutral buffered formalin. Fixed lungs were paraffin embedded into 5μm sections. Sections were stained with hematoxylin and eosin and visualized on the DynamyxTM digital pathology platform. Lungs were scored for chronic and acute inflammation in the lung parenchyma, blood vessels, and airways by a blinded pathologist. Pulmonary inflammation was quantified by measuring the total area of lung tissue involved by inflammation. The predominant inflammatory cell type was noted. To quantify vascular margination of inflammatory cells, five representative arteries were identified within areas involved by inflammation. Total length of endothelium of these vessels was measured and the number of marginating inflammatory cells in the cross section were manually counted. Areas involved by inflammation were further evaluated by electron microscopic examination. The areas of interest were punched from the paraffin embedded tissue block and processed for electron microscopy. Ultrathin sections were cut on a Leica Ultra-Microtome, collected on copper mesh grids, stained using uranyl acetate and lead citrate and viewed using a Jeol 1010 electron microscope (FEI) with attached AMT camera.

### Illumina library preparation, sequencing, and *in silico* bioinformatic analysis

After purification, the concentration of RNA in each sample was quantified using Qubit 3.0 Fluormeter using the RNA high sensitivity (Life Technologies) kit. RNA integrity was assessed using an Agilent TapeStation by Admera Health (South Plainfield, NJ). Illumina sequencing libraries were prepared after samples were DNAsed with KAPA RNA HyperPrep Kit with RiboErase (Basel, Switzerland). The resulting libraries passed standard Illumina quality control PCR tests and were sequenced using the Illumina NovaSeq s4 4000 at Admera Health. A total of ~100 million 150 base pair reads were acquired per sample. Sequencing data will be deposited to the Sequence Read Archive. The reads were trimmed for quality and mapped to the *Mus musculus* reference genome using CLC Genomics Version 21.0.5. Two mice were excluded due to no detectable viral reads (n = 5 NC, 4 Alpha Day 2, 5 Alpha Day 6, 5 Delta Day 2, 4 Delta Day 6). An exported gene expression browser table is available upon request. Statistical analysis of read data was performed with CLC’s Differential Expression for RNA Seq tool and genes were annotated with reference mouse gene ontology terms. Quantification of the number of activated or repressed genes unique to each experimental group was performed using Venny 2.1 [69]. Genes from each experimental comparison with significant fold changes (Bonferroni ≤ 0.04) were submitted to the WEB-based Gene SeT AnaLysis Toolkit’s Over Representation Analysis (ORA) software compared to the reference set “affy mg u74a” to determine GO terms from gene ontology and biological process databases (FDR ≤ 0.05) [70]. GO Term heat maps were generated using Morpheus [71]. Raw read data is available at NCBI SRA: PRJNA808079. An expression browser containing fold change and total counts data has been updated to Mendeley Data [72].

### Statistical analyses

The statistical comparisons made in these studies were calculated using GraphPad Prism version 9. Statistical analyses were performed with n ≥ 3 for all K18-ACE2 mice studies. Comparisons of normally-distributed data sets were made using ordinary one-way ANOVA with Dunnett’s multiple comparisons test. Kruskal-Wallis with Dunn’s multiple comparisons tests were used to assess non-parametric distributed datasets. Kaplan-Meier survival curves were created, and Log-rank (Mantel-Cox) tests were used to test the significance of survival differences between sample groups.

## Supporting information

S1 Table Summary of K18-hACE2 mouse sex and age used in Fig 1. (DOCX)

S2 Table Core activated genes in K18-hACE2 mouse lung after SARS-CoV-2 VOC challenge. (XLSX)

S3 Table (XLSX)

S4 Table (XLSX)

S5 Table (XLSX)

S6 Table (XLSX)
