# Supplementary material for: SARS-CoV-2 Delta variant induces enhanced pathology and inflammatory responses in K18-hACE2 mice
Source: PLoS One. 2022 Aug 29;17(8):e0273430. doi: 10.1371/journal.pone.0273430 (PMC9423646; doi:10.1371/journal.pone.0273430)
Supplement: S1 Table — (DOCX) [file pone.0273430.s001.docx]

SARS-CoV-2 Delta variant induces enhanced pathology and inflammatory responses in K18-hACE2 mice

Lee KS, et al.

**Table S1**

| **Study** | **Variant** | **Challenge Dose** | **Sex** | **Age (at time of challenge** | **Strain** |
| --- | --- | --- | --- | --- | --- |
| Figure 1 1,000 PFU  Comparison | WA-1 | 10^3^ PFU | Male/Female | 8 weeks | B6.Cg-Tg(K18-ACE2)2Prlmn/J |
|  | Alpha (B.1.1.7) | 10^3^ PFU | Female | 12 weeks | B6.Cg-Tg(K18-ACE2)2Prlmn/J |
|  | Beta (B.1.351) | 10^3^ PFU | Male/Female | 8 weeks | B6.Cg-Tg(K18-ACE2)2Prlmn/J |
|  | Delta (B.1.617.2) | 10^3^ PFU | Female | 13 weeks | B6.Cg-Tg(K18-ACE2)2Prlmn/J |
| Figure 1 10,000 PFU  Comparison | WA-1 | 10^4^ PFU | Female | 17 weeks | B6.Cg-Tg(K18-ACE2)2Prlmn/J |
|  | Alpha (B.1.1.7) | 10^4^ PFU | Male/Female | 8 weeks | B6.Cg-Tg(K18-ACE2)2Prlmn/J |
|  | Beta (B.1.351) | 10^4^ PFU | Male/Female | 8 weeks | B6.Cg-Tg(K18-ACE2)2Prlmn/J |
|  | Delta (B.1.617.2) | 10^4^ PFU | Female | 20 weeks | B6.Cg-Tg(K18-ACE2)2Prlmn/J |
| Alpha / Delta Comparison | Alpha (B.1.1.7) | 10^3^ PFU | Male | 8 weeks | B6.Cg-Tg(K18-ACE2)2Prlmn/J |
|  | Delta (B.1.617.2) | 10^3^ PFU | Male | 8 weeks | B6.Cg-Tg(K18-ACE2)2Prlmn/J |
